# Approaching sleep apnea management in the setting of uncertainty

**DOI:** 10.1093/sleep/zsac234

**Published:** 2022-09-23

**Authors:** Lucas M Donovan, Sanjay R Patel

**Affiliations:** Division of Pulmonary, Critical Care, and Sleep Medicine, University of Washington and Veterans Affairs Puget Sound Health Care System, Seattle, WA, USA; Center for Sleep and Cardiovascular Outcomes Research, University of Pittsburgh, Pittsburgh, PA, USA

Medical decisions are best made in consultation with high-quality evidence, but, where such evidence is incomplete, clinicians must counsel patients using available knowledge. This is the case for patients with obstructive sleep apnea (OSA) who lack excessive daytime sleepiness (EDS) and established cardiovascular disease (CVD). In the current issue of *SLEEP*, Dr. Pengo and colleagues offer a framework for management that contextualizes current evidence [1]. The authors propose the three-step OSCAR algorithm where clinicians sequentially address excess weight, assess symptoms, and consider cardiovascular risk. While we believe that this algorithm is logical and helpful, there are nuances that merit consideration.

In its first step, the OSCAR algorithm advises referring patients with overweight or obesity to weight loss clinics for behavioral interventions aimed at achieving 10% weight loss. We agree with this approach, although implementation will be challenging. Despite longstanding recommendations that patients with OSA are counseled about weight loss and referred for services, few patients receive evidence-based weight loss interventions [2]. Even in a health system with an integrated weight loss program, only a small fraction of patients receive these services [3, 4]. Novel approaches such as telemonitoring of lifestyle modifications show promise in OSA [5], but additional strategies are needed. While the OSCAR algorithm specifically recommends lifestyle modifications, these alone are often insufficient to achieve 10% weight loss [2]. Additional treatments should be considered, particularly GLP-1 agonists that have been established to reduce cardiovascular event rates [6, 7]. Sleep clinicians should familiarize themselves with the full spectrum of weight loss options to achieve meaningful weight loss.

The OSCAR algorithm then suggests that clinicians reassess symptoms. The authors raise two distinct issues: (1) reliance on the Epworth Sleepiness Scale (ESS) score may underdiagnose EDS, and (2) additional symptoms beyond EDS may be worth treating. Given the strong evidence that CPAP improves EDS, it is important that we do not miss this symptom. We fully endorse the authors’ recommendation to include family members in assessing symptoms and to use a holistic approach in evaluating EDS. While the ESS is the instrument most often used to assess sleepiness [8], it has notable limitations including poor reproducibility and lacks validation in important subgroups [9, 10]. Future work needs to identify the optimal instruments to diagnose and monitor EDS over time. The authors suggest the use of objective measures (e.g. multiple sleep latency testing) when clinicians suspect EDS despite a normal ESS. While objective testing has its merits, we doubt the sustainability of routine use. Objective tests are resource intensive and require substantial time investment from patients. In addition to EDS, the authors propose that clinicians assess 14 nocturnal (e.g. insomnia) and diurnal (e.g. difficulty concentrating) symptoms that have been attributed to OSA. However, unlike EDS, we lack strong evidence from double-blind randomized trials that CPAP can improve these symptoms [8]. While these symptoms may improve with CPAP, we need to rigorously test these assumptions.

Among patients without symptoms, the OSCAR algorithm then considers cardiovascular risk. The authors propose evaluating OSA-induced cardiovascular risk by employing ambulatory blood pressure monitoring and prescribing CPAP to patients with hypertension, particularly if there is a non-dipping pattern. This decision rests on evidence that CPAP reduces blood pressure and the association of hypertension and non-dipping patterns with cardiovascular risk [11]. Among asymptomatic patients with normal blood pressure, the authors further suggest that clinicians consider CPAP use in those with excessive physiologic disruptions (in terms of hypoxemia or sleep fragmentation) believed to causally contribute to cardiovascular risk. This proposal assumes that CPAP will have the greatest benefit in those with intermediate phenotypes that are presumed to cause CVD. However, in selecting patients for statin prescription, a global CVD risk assessment is more effective than one focused on mechanistic pathways (i.e. lipid levels) [12]. Thus, it will be important to compare OSCAR’s approach with an alternative strategy of selecting patients for CPAP who are at the greatest overall CVD risk.

As we consider the potential impact of the OSCAR algorithm on CVD, we must consider the challenge posed by primary prevention. The OSCAR algorithm focuses on primary prevention because previous trials among patients with preexisting CVD and low symptom burden failed to find a cardiovascular benefit with CPAP [8]. However, we must be aware that even if CPAP has a salutary effect for primary prevention, the absolute benefit is likely to be low. For example, although a recent observational cohort found a greater association between CPAP use and cardiovascular risk reduction for primary versus secondary events on a relative scale, due to the much lower baseline event rate in those without existing CVD, the absolute risk reduction was 10-fold lower for the primary prevention group [13, 14]. Thus, the cost–benefit assessment of CPAP for primary prevention will likely be unattractive to many patients, health systems, and payors.

A further complication relates to adherence. Many attribute the negative results for CPAP in secondary prevention trials to low adherence [15, 16]. Since step 3 of the OSCAR algorithm focuses on patients carefully selected to be asymptomatic, we should anticipate similar difficulties while implementing the OSCAR algorithm. Furthermore, without prior cardiovascular events to reinforce the salience of preventive treatments, adherence will likely be even lower. While strategies to enhance adherence are available, we must be prepared for the costs and complexity that such co-interventions would entail. When counseling patients, we must also consider the patient burdens of adherent CPAP use (e.g. mask discomfort, cleaning) relative to the likelihood of CVD benefit.

Finally, one must consider the potential consequences of deploying this algorithm within a health system. In designing the OSCAR algorithm, the authors focused on decisions that are justifiable at the individual patient level and the authors appropriately acknowledge the principle “primum non nocere”—first do no harm. However, it is important to look beyond the individual when considering harm. Sleep medicine services are a scarce resource [17, 18], and limited access leaves millions worldwide undiagnosed, untreated, or treated without appropriate follow-up. When a treatment is scarce, the most ethical choice is to allocate that treatment in a way that maximizes benefit [19]. By establishing pathways that cater to patients for whom we lack ­high-quality evidence of benefit, we may divert resources away from fully treating patients for whom we *do* have high-quality evidence of benefit (e.g. excessive daytime sleepiness). Furthermore, evidence suggests that those currently least likely to receive effective OSA care are those marginalized by society—individuals who are poor, with low health literacy, and from minority backgrounds [20–24]. As a field, we certainly need to implement evidence-based strategies that improve our capacity to meet needs (e.g. home testing, alternate care providers). However, we also need to be conscious of our scarce resources and ensure that we do not unintentionally widen sleep health disparities by offering treatments without benefit to those with privilege and the ability to demand care.

Despite its potential limitations, the OSCAR algorithm is a welcome addition to the literature. As the authors clearly point out, it is a proposal that needs to be rigorously tested. By providing a clear and logical framework, the OSCAR algorithm identifies specific areas of uncertainty in the clinical management of OSA in those without EDS or CVD. OSCAR also serves as an excellent starting place for future trial protocols to resolve uncertainties around how to manage this important patient population.
